# Models for an Ultraviolet-C Research and Development Consortium

**DOI:** 10.6028/jres.126.055

**Published:** 2022-03-25

**Authors:** Dianne L. Poster, Michael T. Postek, Yaw S. Obeng, John J. Kasianowicz, Troy E. Cowan, Norman R. Horn, C. Cameron Miller, Richard A. Martinello

**Affiliations:** 1 National Institute of Standards and Technology, Gaithersburg, MD 20899, USA; 2 University of South Florida, Tampa, FL 33612, USA; 3 Columbia University, New York, NY 10027, USA; 4 International Ultraviolet Association, Bethesda, MD 20815, USA; 5 Seal Shield, LLC, Orlando, FL 32801, USA; 6 Yale School of Medicine and Yale New Haven Health, New Haven, CT 06510, USA

**Keywords:** capacity building, collaboration, disinfection, hospitals, innovation, market growth, partnerships, pathogens, public health, ultraviolet, UV-C, viruses

## Abstract

The development of an international, precompetitive, collaborative, ultraviolet (UV) research consortium is discussed as an opportunity to lay the groundwork for a new UV commercial industry and the supply chain to support this industry. History has demonstrated that consortia can offer promising approaches to solve many common, current industry challenges, such as the paucity of data regarding the doses of ultraviolet-C (UV-C, 200 nm to 280 nm) radiation necessary to achieve the desired reductions in healthcare pathogens and the ability of mobile disinfection devices to deliver adequate doses to the different types of surfaces in a whole-room environment. Standard methods for testing are only in the initial stages of development, making it difficult to choose a specific UV-C device for a healthcare application. Currently, the public interest in UV-C disinfection applications is elevated due to the spread of severe acute respiratory syndrome coronavirus 2 (SARS-CoV-2), the virus that causes the respiratory coronavirus disease 19 (COVID-19). By channeling the expertise of different UV industry stakeholder sectors into a unified international consortium, innovation in UV measurements and data could be developed to support test methods and standards development for UV healthcare equipment. As discussed in this paper, several successful examples of consortia are applicable to the UV industry to help solve these types of common problems. It is anticipated that a consortium for the industry could lead to UV applications for disinfection becoming globally prolific and commonplace in residential, work, business, and school settings as well as in transportation (bus, rail, air, ship) environments. Aggressive elimination of infectious agents by UV-C technologies would also help to reduce the evolution of antibiotic-resistant bacteria.

## 1 Introduction

**Fig. 1:**
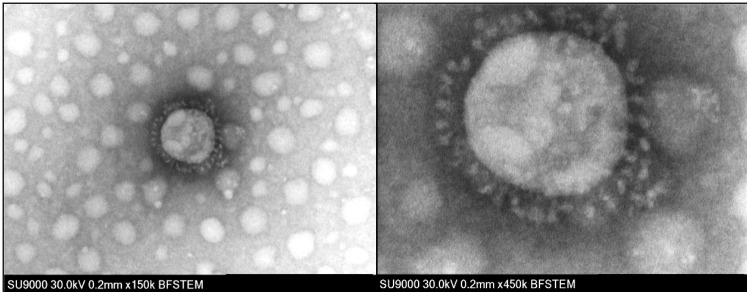
Bright field scanning transmission electron micrograph (BF-STEM) of the coronavirus using a Hitachi SU9000. (Left) Coronavirus shown is about 150 nm in diameter (field of view ≈ 845 nm). (Right) Smaller field-of-view image of the coronavirus showing the spike proteins (field of view ≈ 280 nm). Images are courtesy of Hitachi High-Tech Corporation, personal communication, Michael Postek.

**Fig. 2:**
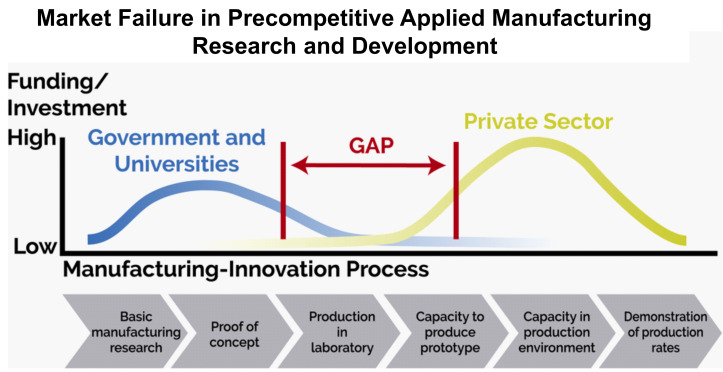
Bridging the metaphorical “valley of death,” the gap between an idea and a product in the manufacturing landscape, requires more than innovation. Integration of success factors, such as science, personnel, intellectual property, infrastructure, and capital, is necessary, along with shared solutions for broad industry challenges that no one can do, or do alone, because of risk or lack of capacity [9]. Standards are essential along the manufacturing-innovation process to ensure the quality and comparability of the product in the production environment, and beyond, with other products on the market. Standards also ensure customers ultimately receive a product that will perform as expected. Figure credit: National Institute of Standards and Technology (NIST).

A pathogen is a bacterium, virus, or other microorganism that can cause disease that can lead to death. Pathogens cause healthcare-associated infections (HAIs), which are recognized as serious public health and patient safety problems. Ultraviolet-C (UV-C, 200 nm to 280 nm) devices have been shown to decrease the risk for HAIs via treatment of surfaces in the healthcare environment known to carry, transmit, or sustain pathogens [1, 2]. Despite this logic and the demonstrated applicability of UV technology to treat and prevent pathogen spread, its use in the healthcare industry has been sparse and uneven [3]. However, there is interest in adopting the technology for better public health, particularly due to the spread of severe acute respiratory syndrome coronavirus 2 (SARS-CoV-2) [4], which causes coronavirus disease 19 (COVID-19) (Fig. 1), and other communicable diseases. Currently, a lack of uniform performance standards for measures of UV-C biological efficacy in public health applications makes innovation and market growth difficult [5, 6] (Fig. 2). Uniform performance standards are essential to enable healthcare managers to make informed, credible investment decisions about any UV device for infection control and prevention, especially as new technologies are being developed with wavelengths other than 254 nm [6].^1^1 The use of UV technologies for public health is different from UV for water disinfection. See the following references for more information on UV water disinfection: Poster *et al.* [6]; Bolton and Cotton [7], and the U.S. Environmental Protection Agency [8]. The International Ultraviolet Association also has guidance for wastewater UV validation applications (see Table 2 in Sec. 2.2 below).

Commercialization of products using new or evolving technologies is often surrounded by significant research and investment needs that must be overcome to propel the technology, even when it has proven efficacy, low risk, and high yield (Fig. 2) [9]. No single company can solve the needs for an entire industry, particularly when fundamental measurement science (*i.e.*, metrology) gaps must be solved. Historically, industry-driven collaborative models, such as consortia, have successfully developed durable and scalable technical solutions that advance an industry. An example is SEMATECH,^2^2 SEMATECH is an acronym for Semiconductor Manufacturing Technology [10]. which began in 1987 as a government-industry collaboration to develop technologies in the semiconductor industry using a combination of industry and federal government funding [10]. Its initial membership included 14 firms constituting 80% of the U.S. semiconductor manufacturing industry, and it evolved to become International SEMATECH—a collaboration of private companies. SEMATECH and other examples of collaborative models are discussed in Sec. 3 in the context of the UV industry and UV-C technologies for decreasing surface contamination and reducing HAIs. Given the size and impact of the international UV disinfection market [11], and consideration of the emergence of COVID-19 [4, 6, 11], it is possible that a UV industry consortium for healthcare applications could be similar in size to the original SEMATECH consortium.

The reduction of HAIs has consistently been a top priority for the U.S. Centers for Disease Control and Prevention and its partners in public health and healthcare, with the goal to prevent and eliminate HAIs to the point where they are considered “rare, unacceptable events” [12]. In the presence of the COVID-19 threat, there is a greater incentive for industry to focus attention on reducing HAIs. An examination of the total number and economic impacts of HAIs in the United States helps to convey the long- and near-term importance of the UV industry in healthcare. In 2011, 4% of hospitalized patients were reported as being affected by one or more HAIs in acute care hospitals [13]. In 2015, this decreased to 3.2% [14]. The deaths from HAIs have also been decreasing, for example, from approximately 98,987 deaths in 2002 [15] to about 72,000 in 2015 [16]. These reductions can be attributed to the enhanced implementation of prevention interventions and strategies [14]. Recent research suggests HAI reduction increases revenues for hospitals by a significant margin while improving patient well-being and saving lives [17].

Even with demonstrated progress in reduced HAI cases and deaths, more work remains to prevent and eliminate HAIs to the point where they are considered rare. Here, UV-C can play an important role. However, gaps in the physical and biological sciences remain, and the effectiveness of UV-C devices has been difficult to determine. Data regarding the doses of UV-C necessary to yield desired reductions in healthcare pathogens and the ability of mobile disinfection devices to deliver adequate doses to various surfaces in patient rooms are limited [3]. In addition, variations in UV-C radiation source design (mobile or stationary), capabilities, and application environments make choosing a UV device potentially confusing.

Standard methods for testing devices with standard reporting of doses as a function of reduced inactivation data would greatly alleviate the challenges in choosing a UV device for HAI applications. Standards provide a sense of certainty and confidence to buyers and users of equipment, thus making it easier to make decisions about what, when, and even how to buy. However, the development of standards for UV devices and HAI applications is nascent and limited, as described at the National Institute of Standards and Technology (NIST) workshop, “Ultraviolet Disinfection Technologies and Healthcare Associated Infections: Defining Standards and Metrology Needs,” which took place in January 2020 at the NIST campus in Gaithersburg, MD [6].

A collective effort is needed to build a consensus on standards development now, at the onset of their development. Frieden [12] noted specifically that to achieve a new normal where HAIs are considered rare events in healthcare, multidisciplinary and multisectoral partnerships are critical. New partners bringing new ideas and opportunities for HAI prevention, while working alongside traditional partners in the field, are essential to strengthen proven UV-C prevention strategies and advance research aimed at addressing knowledge gaps [12].

Several partnership models, illustrated with examples of engagement among industry, government, and academia, are discussed here. There is no universal strategy for partnership development. An example consortium model is provided in Sec. 5 as a possible next step toward achieving greater public health, security, and economic strength through UV disinfection technology, which were key targets identified at the NIST workshop [6]. Since the workshop, the International Ultraviolet Association (IUVA) has begun exploring the establishment of a task force to study the development of a chartered consortium to support advancement of UV disinfection technologies for healthcare applications through industry partnerships.

## 2 UV for Healthcare-Industry Partnerships

### 2.1 Community Engagement with NIST on UV for Healthcare

The NIST workshop was a full-scale, two-day, international workshop hosted in collaboration with the IUVA and its members and affiliates [6]. Throughout the event, targeted discussions took place on metrology requirements needed to advance UV-C engineering and operations for healthcare, HAI biology measurements and characterization techniques to support efficacy measures, and regulatory issues surrounding disinfection for public health. Discussions converged to identify the best available and required measurements, standards, and data in the context of innovation and the effective use of UV-C and other radiation spectra for applications in healthcare and their implementation. UV device manufacturers and their stakeholders (Table 1) share interest in and responsibility to create conditions for the safe, stable, and sustainable operations of UV devices in healthcare settings. Table 1 demonstrates the breadth and depth of UV stakeholders based on UV community engagement efforts described by Poster *et al.* [6].

During the NIST workshop [6], it was suggested that the goals of the UV industry could be facilitated by the development of a precompetitive collaborative research consortium. Such an organization could offer an approach to greatly utilize and leverage the broad-based subject matter expertise and domain authority available within the UV stakeholder community, expertise that, if pooled into a research collaboratory (or collaboratories), could overcome many of the overarching issues described above in Sec. 1. A *collaboratory* is a place or facility where complementary parties are brought together to collaborate or is an organization that manages such a facility. While a physical location is not necessary for a research collaboratory, a number of coordinated collaboratories at universities or member companies could be established, each with its own expertise, working for the common goals of the consortium and all belonging to the overarching consortium.

An international consortium could be noncompetitive and work toward the good of all the members to further market growth in UV technology applications. Under this model, the member companies would quickly learn that they are independent but have common problems that can be resolved within the consortium. These needs, once identified, would be rooted across the spectrum as infrastructural, not competitive. Current examples include the paucity of data regarding the doses of UV-C necessary to achieve the desired reductions in healthcare pathogens and the ability of mobile devices to deliver adequate doses to the different types of surfaces in a whole-room environment [3]. These data are needed by all members of the UV industry. In addition, UV-C technology has been adopted broadly by the disinfection market for clean water access and distribution across the globe, with a current estimated $4.8 billion market that enables greater public health via elimination of pathogens responsible for waterborne diseases [11]. Utilizing a consortium approach, this significant expertise and market domain could be leveraged to help develop such global reach efforts for the treatment and prevention of pathogens in healthcare settings. For example, consortium projects targeted toward developing UV dose data to support efficacy standards for testing the delivery of doses to surfaces by UV mobile devices would be a good beginning.

**Table 1:** Ultraviolet industry stakeholders.

Stakeholder	Examples
Industry	Lamp and device manufacturers Sensor and calibration equipment manufacturers
Industry	Air, surface, and water, including wastewater, disinfection companies Industrial hygiene, housekeeping, and janitorial service providers (environmental services) Healthcare facility air, water, and physical infrastructure operation providers
Industry	Testing, validation, and biological science companies (research and development [R&D] and supply) Quality assurance and quality monitoring companies
Industry	Mass transit (bus and rail) and private transportation (air, motor, rail, ship) entities Transportation terminal operations and service providers Public and private education and daycare infrastructure
Healthcare providers and specialists and their organizations; academic researchers and their institutions	Physicians, nurses, ambulatory care specialists, and hospital administrators Healthcare epidemiologists, information specialists, clinical microbiologists, and infection prevention specialists Medical, life science, and engineering researchers Organizations, such as hospitals and medical research centers Academic institutions
Healthcare initiative experts and authorities and their supporting organizations or academic institutions	Antibiotic stewardship, pharmacy, and therapeutic entities Clinical microbiology laboratories Quality assurance and patient safety units Organizations, such as hospitals and medical research centers Academic institutions
Technical and industry support organizations	Trade specialty groups and industry associations, including the IUVA Standards development organizations (see Sec. 2.2 below)
Federal agencies	Agencies with public health and safety, transportation, and consumer products oversight Agencies with research programs in the physical, chemical, and biological sciences
Public and private sectors	Patients and users and employees of public systems, such as education and daycare infrastructure, and businesses open to the public, such as grocery stores and restaurants employees of private-sector companies, including offices

### 2.2 Community-Wide Standards Development for UV Healthcare Applications

Recent actions suggest that stakeholders (see Table 1) are motivated to participate in a partnership model. Calls-to-action have been considered [18], and the IUVA has already made progress on developing, updating, and publishing guidance material (Table 2) [19]. For UV healthcare applications, NIST has engaged with the community on measurements, standards, technology, and data [6]. Moreover, a roadmap plan for standards supporting UV healthcare applications has been developed through the IUVA healthcare working group (Table 3), in which NIST participates. However, the working group is not a consortium, but rather a group of representatives from the UV public and private sector communities coordinating and sharing information. While there are currently more than 100 members, the working group does not fund shared projects like a formal consortium would do.

**Table 2:** IUVA guidance documents to date [19].

Name	Brief Description
Protocol for the determination of fluence (UV dose) using a low-pressure or low-pressure–high-output UV lamp	This protocol is based on the paper by Bolton and Linden [22], but it is set out in a step-by-step manner to make it easier to follow for experimental measurements [23].
Method for the measurement of the output of monochromatic (254 nm) low-pressure UV lamps	This protocol was developed to present a consistent methodology for the determination and benchmarking of UV lamp output from monochromatic (254 nm) lamps operated by a corresponding power supply (ballast) [24].
Fluence (UV dose) required to achieve incremental log inactivation of bacteria, protozoa, viruses, and algae	This protocol represents the second revision of a compilation that goes back to 1999 [25] and was an internal document of Trojan Technologies. An updated version of this paper was published in a special section of the *Journal of Research of the National Institute of Standards and Technology* on UV disinfection technologies [26].
Uniform protocol for wastewater UV validation applications	This protocol represents uniform wastewater protocol and includes protocol for planning and preparation, microbiological testing, validation data analysis, additional analysis using advanced tools and existing data, and reporting [27].

The IUVA healthcare working group roadmap plan in Table 3 puts in place a vision for UV healthcare applications supported through standards development. This is an international effort. Member companies within IUVA and other organizations participate from the United States, Canada, Australia, Singapore, Japan, China, Turkey, Israel, many countries in the European Union, and beyond. In addition, the standard-development infrastructure of the international lighting community is being leveraged [20]. As standards are developed, U.S. and international companies will be able to maximize their planning and forecasting capabilities. Standard test methods will ensure a reliable consistent market through measurement capacity building. For example, once test methods are established, third-party laboratories can invest in equipment and capabilities to support the disinfection industry. The laboratories then can become accredited, which means an accreditation body like the National Voluntary Laboratory Accreditation Program can access laboratories to assess their management and quality systems against well-established, globally accepted processes as described in the U.S. Standards Strategy (USSS) [21].

The USSS [21] outlines the roles of international standards development organizations, such as the American National Standards Institute (ANSI), the coordinator of the U.S. private-sector voluntary standardization system and the official U.S. representative in regional and nontreaty international standards development bodies; the International Organization for Standardization (ISO); and, via its U.S. National Committee, the International Electrotechnical Commission (IEC). The USSS recognizes the importance of international engagement for standards development and the need to strengthen participation by government at all levels in the development and use of voluntary consensus standards through public-private partnerships.

**Table 3:** UV standards roadmap plan—A proposed scope.^a^

Name	Plans
1. Standard for measuring UV lamp and luminaire irradiance	1.1. Gaseous discharge lamps (Hg, xenon, continuous & pulsed) 1.2. Light-emitting diodes (continuous & pulsed) 1.3. Excimers and lasers
2. Standard for measuring UV luminaire antimicrobial efficacy	2.1. Surface (two-dimensional and three-dimensional; *e.g.*, whole-room devices, cabinet enclosures, handheld devices, mobile devices) 2.2. Air (*e.g.*, heating, ventilation, and air conditioning [HVAC] internal, upper air, portable air filter UV devices) 2.3. Water (*e.g.*, utility system treatments such as potable water and municipal wastewater), building system treatment (*e.g.*, commercial, residential), agriculture, and point of use
3. Standards for calibrating UV irradiance measurement systems	3.1. Radiometers—intensity of radiant energy 3.2. Dosimeters—industrial and personal needs 3.3. Other
4. Standards for measuring UV transmittance and absorption	4.1. Gaseous—air applications 4.2. Liquid—water applications 4.3. Solid—surfaces, matter interaction applications
5. Standards for measuring UV dose response curves and action spectra	5.1. Pathogen specific & specific wavelengths (*e.g.*, 222 nm, 254 nm, 265 nm, 405 nm) 5.2. Pathogen specific across the continuous UV spectrum (from 150 nm to 400 nm or more)
6. Standards guidance and reports	6.1. To be determined
7. Terminology and reporting	7.1. Glossary of UV standards terminology 7.2. Harmonization of reporting
8. Data hub	8.1. Directories of materials (standards, guidance, best practices, *etc*.) that are proposed, in progress, or completed

^a^ Developed with input from internationally engaged organizations through the IUVA healthcare working group, including the American Society of Heating, Refrigerating and Air-Conditioning Engineers (ASHRAE); International Commission on Illumination (CIE); International Organization for Standardization (ISO); ASTM International; Association for the Advancement of Medical Instrumentation (AAMI); and Underwriters Laboratories (UL).

A UV industry consortium should actively pursue the development of standards in collaboration with standards development organizations to produce standards that have an industry-wide impact and that meet the federal definition of “voluntary consensus standards” as contained in Office of Management and Budget (OMB) Circular A-119 “Federal Participation in the Development and Use of Voluntary Consensus Standards and in Conformity Assessment Activities,” thus alleviating the need for most, if not all, federal regulations covering the same or similar requirements [28]. Specifically, OMB A-119 requires “...all Federal agencies must use voluntary consensus standards in lieu of government-unique standards in their procurement and regulatory activities, except where inconsistent with law or otherwise impractical [28].” This is beneficial because it eliminates the cost of developing similar federal standards (where industry’s “voluntary consensus” standards already exist) and decreases the cost of goods procured and the burden of complying with agency regulations, while assisting the federal agencies in reducing regulatory requirements in conformance with Executive Orders 13563 and 13610, “Identifying and Reducing Regulatory Burdens.”

## 3 Consortium Models in Technology Industries

### 3.1 Models that Include the U.S. Federal Government

**Fig. 3:**
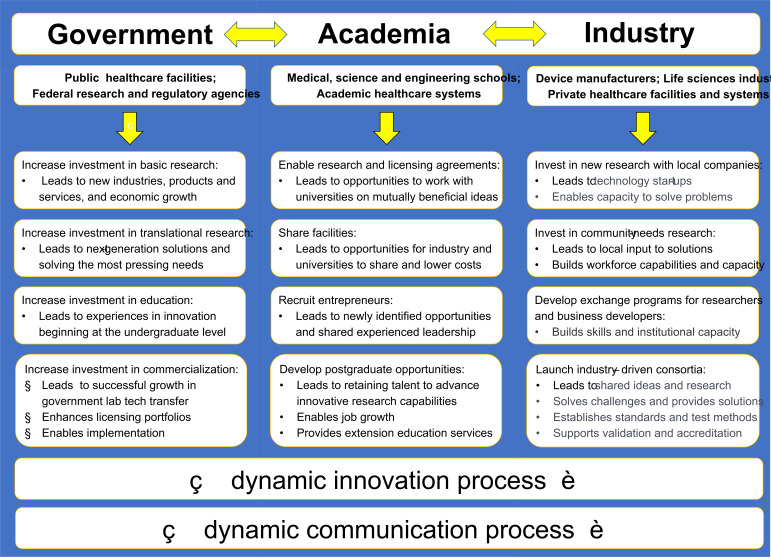
Primary needs to be considered in partnerships for a dynamic innovation process that leads to lasting effects in an innovation-driven economy, adapted from Young [29] and Pakes and Sokoloff [30]. The U.S. economic vitality requires a substantial investment in research and development that is leveraged through government, university, and industry partnerships, with consortia being a cornerstone (bottom right) to make this possible. For example, the National Aeronautics and Space Administration (NASA) provides opportunities for partnerships so that businesses can utilize NASA’s capabilities and resources to further their capabilities and NASA’s missions.^3^3 See https://www.nasa.gov/partnerships.html for more information on NASA partnerships. A factor equally important to the dynamic innovation process is a communication process that engages all entities. See Sec. 5.3 for a discussion on the importance of communication and perception strategies in partnership models supporting new technologies.

In the United States, there are several precompetitive collaboration models involving the federal government that have had demonstrated success. Participation of the government in a consortium model with academia and industry enables a wider range of roles and responsibilities for each entity (Fig. 3). Young [29] reasoned that such roles and responsibilities ensure the successful development of commercial products and services and that, by collaborating dynamically, consortia become the cornerstone to sustained success (Fig. 3, bottom right). For example, for the UV industry, basic research in photochemistry and photobiology and standards research and education would be supported by the government (see the first three boxes under “government” in the left column of Fig. 3). The benefits from these investments would dynamically support academic successes through more advanced research and engagement with industry, which in turn would support additional advances in academia and industry through community growth and the use of applications by end users.

Collaborative models that engage the federal government sometimes start with federal interagency working groups. For example, in the field of quantum information science (QIS), interagency working groups have enabled coordination of transformational technological advances between federal and nonfederal entities using a strategic approach to bring together potential partners for collaboration. Information is shared across disciplines, and hard technical problems are identified and prioritized with the help of a National Quantum Coordination Office^4^4 See www.quantum.gov for more information on coordinated efforts to advance QIS, including those with industry in the United States and around the globe. and various working groups (Table 4) [31]. Working groups foster an environment for further information sharing among more partners with a focus on technology development and commercialization. This coordinated approach helps agencies to stay abreast of new technology and products and the ways in which they may affect their missions, enables industry engagement, provides mechanisms for public-private partnerships, and offers access to international cooperation and resources [32].

**Table 4:** U.S. National Science and Technology Council Quantum Information Science Subcommittee coordination entities [31].

Entity for Coordination	Brief Description of Role
National Quantum Coordination Office	Developing and supporting mechanisms that enhance and sustain collaboration
Science working groups	Coordinating the scientific and technical aspects of programs
Workforce, infrastructure, and industry working groups	Identifying workforce and technology needs and developing coordinated recommended solutions
End-user working groups	Connecting the nation’s research and development community, including academics and industry participants, to potential early adopters

**Fig. 4:**
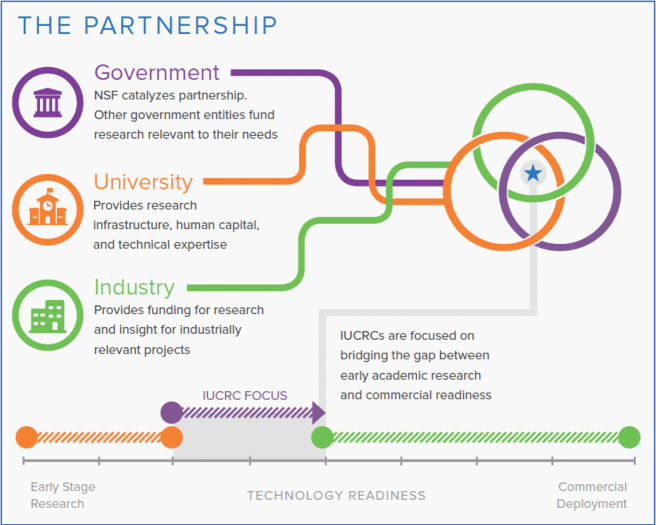
The Industry-University Cooperative Research Center (IUCRC) consortium model, demonstrating the IUCRC’s primary role in translating research from concept to commercialization. Credit: National Science Foundation [33]. Industry, in green, provides funding for research and insight for industrially relevant projects and helps lead to commercialization of projects, as shown in the bottom portion of the figure in green on the time line of technology readiness.

Another example of a collaborative model that engages the federal government is an industry-driven cooperative research center connected to a university through Industry-University Cooperative Research Centers (IUCRC) (Fig. 4). These are specifically created to catalyze precompetitive research in areas of strategic interest to U.S. industry. These are funded through the U.S. National Science Foundation (NSF) Industrial Innovation and Partnerships Program. Centers are university-based industrial consortia.

IUCRC members provide financial support, help steer research, and share results [33, 34]. The program has initiated more than 140 centers. IUCRCs were designed to serve as institutional structures to promote and sustain scientific cooperation between industry and universities. They have been successful in achieving this objective for more than three decades, and this model represents the longest operating partnership-based program sponsored by NSF. Industry partners join at inception and can start new developments with other universities in parallel to leverage NSF support. Government agencies participate as members or by partnering directly with NSF at the strategic level. Industry participants can be major corporations, middle market companies, small businesses, and startups. Government participants may range from local governments to divisions of federal agencies. A membership fee structure supports research projects conducted by university faculty and students. The NSF provides funding for operations and a governance framework for membership, operations, and evaluation. This is an example of a modern public-private partnership.

Both examples shown here enable resources to be distributed and applied to the difficult problems all members of a partnership face at a precompetitive level. In addition, the entire membership has a voice and establishes legitimacy that can be leveraged for funding opportunities. Another example of this scenario is the NIST Advanced Manufacturing Technology Consortia (AMTech) program [35]. While this program is no longer active, it was a competitive grants program created to establish new or strengthen existing industry-driven consortia that address high-priority research. The program enabled many successful technology roadmaps that helped to build the current Manufacturing USA network. In addition, five Manufacturing USA institutes were created and are still in operation today: AIM Photonics in Albany, NY (integrated photonics); MxD in Chicago, IL (digital manufacturing); LIFT in Detroit, MI (lightweight materials manufacturing); NIIMBL in Newark, DE (biopharmaceutical manufacturing); and REMADE in Rochester, NY (sustainable manufacturing). AmTech also helped to determine the research projects that these and other institutes are currently pursuing. Such an approach could be a model for the UV industry, *i.e.*, as an international network of partners with regional- or country-specific hubs.

Another example of government partnership models is the current BRAIN Initiative Alliance. This alliance is a self-assembled partnership populated by federal and nonfederal members and affiliates to “accelerate the development and application of new technologies that will enable researchers to produce dynamic pictures of the brain that show how individual brain cells and complex neural circuits interact at the speed of thought” [36].

In response to the grand challenge posed by the alliance, the U.S. National Institutes of Health (NIH) convened a working group with over 75 experts to later launch the “Brain Research through Advancing Innovative Neurotechnologies” (BRAIN) Initiative [37]. This initiative is producing new technologies through funding opportunities that are the result of the alliance model. An example of success is the development of new approaches to measure and map how individual cells and complex neural circuits interact in both time and space. The industry-driven alliance model is a cornerstone consortium approach as called for by Young [29] (see Fig. 3). It enables a dynamic innovation process that is sustainable through its revolutionary technology developments, products, and end-use of those products by the communities the alliance supports. There are numerous funding opportunities within the BRAIN Initiative Alliance that are continuously updated and open to the public [38], with opportunities ranging from proof of concept to small business technology transfer.

Nonprofit foundations are also beginning to support research at federal agencies through actively seeking and receiving monetary donations from private donors and organizations to support their missions. These arrangements can be congressionally mandated, such as the Foundation for the National Institutes of Health (FNIH), which can raise nonfederally appropriated funds and generate revenue to reinvest in research and development. In 2019, the total revenue and support for FNIH was over $56 million, with about 89% of that supporting research and development programs and enabling private partners to substantially expand the number of funded NIH grants. In 2020, the total revenue and support for FNIH almost doubled to $102 million [39].

### 3.2 European Government Collaboration Models for Technology Development

Europe has numerous collaborative fora to enable economic competitiveness and resilience for technology industries in the European Union (EU). Horizon 2020, the EU’s Framework Programme for Research and Innovation (EU-FP), is a prime example. Horizon 2020 is the financial instrument implementing the Innovation Union, a Europe 2020 flagship initiative aimed at securing Europe’s global competitiveness. The EU-FP does this by strengthening the scientific and technological bases of European industry, and by promoting research activities in support of other EU policies through shared resources distributed through project consortia that self-organize, develop proposals, and compete for funding. Members of a consortium can be from outside the European Union. Collaboration choices in the EU-FP are primarily facilitated by prior acquaintance and thematic and geographical proximity [40]. Consortia with high levels of experience and sterling reputation, involving a large share of Western European partners and those engaged in more application-oriented consortia, have been shown to have greater chances of success in acquiring project funding [41].

While the EU-FPs date back to 1984 [40], Horizon 2020 is the biggest EU-FP to date, with nearly €80 billion of funding available from 2014 to 2020, and with additional private investment to augment the program. The three major outputs are: (1) creating networks and integrating research and innovation efforts across countries (154 countries involved), sectors, and disciplines, with 1.5 million one-to-one collaborations in projects; (2) generating scientific breakthroughs, with 62,000 peer-reviewed publications, where three out of five are based on private-sector and/or academia collaboration; and (3) generating innovation, including more than 1600 patent applications, and benefits to society expected from most projects (*e.g.*, Ebola research, climate change, antimicrobial resistance to name a few) [42].

### 3.3 Collaborations Solving Metrology Problems

#### 3.3.1 Industry-Led Examples

As mentioned in Sec. 1, one of the most high-profile examples of a successful, metrology-driven consortium was the International SEMATECH, which inspired other consortia adopted by the semiconductor industry later, some at a much smaller scale [43, 44]. The initial goals of SEMATECH were to unify and define the current and future goals of the industry. Manufacturers, through cooperative roadmapping exercises, strove to meet these goals. In the organization’s early years, it strengthened semiconductor manufacturing equipment suppliers by improving their technical capabilities through funded research [45]. About 80% of its research and development funds supported projects aimed at producing usable results between 12 months and 3 years from the time a project started [10]. By doing so, SEMATECH developed advanced processes to challenge and exercise next-generation manufacturing equipment. These efforts led to the development of the following: new equipment by suppliers; standards for equipment interfaces; improved reliability of manufacturing equipment; qualified new and improved manufacturing equipment for plant application; and improved communications between manufacturers and equipment makers.

A quantitative case-based analysis of the returns to member companies from their investments in SEMATECH suggests that “SEMATECH has provided an organizational structure in which important processes and technologies have been advanced which could not have been justified on economic grounds outside of a collaborative research arrangement” [46]. In other words, the collective efforts helped to overcome challenges that no one single company could solve in isolation. Clearly, through a consortium, the semiconductor industry, rapidly, but incrementally, improved semiconductor manufacturing. The consortium, through its work, was able to change the industry opinion about metrology—initially believing there was “no value added” but changing to “if we cannot measure it, we cannot make it.”

Ultimately, the organization enabled the dissemination of information and best-practice techniques, set standards, and coordinated research, and it has been followed by other U.S. technology development consortia funded from public and private sources [47]. Several successes provided by the founding of SEMATECH are listed in Table 5, including the application of extreme UV (wavelength of 13.5 nm).

**Table 5:** Some of SEMATECH’s key accomplishments [48].

• Laying the groundwork for wafer-size transitions • Establishing and maintaining the semiconductor industry roadmap • Building industry-wide consensus and developing infrastructure for a succession of next-generation lithography technologies, including extreme UV • Guiding the development of robust copper/low-dielectric-constant and three-dimensional interconnect technologies • Receiving the prestigious Climate Protection Award from the U.S. Environmental Protection Agency for work in reducing perfluorocarbon emissions • Establishing the Resist and Materials Development Center and the world’s first Extreme Ultraviolet Lithography (EUV) Mask Blank Development Center, at the College for Nanoscale Science and Engineering (CNSE), University at Albany • Facilitating breakthroughs in advanced device structures and materials, including high-κ metal gate stacks and III-V materials • Launching the U.S. Photovoltaic (PV) Manufacturing Consortium, a partnership between SEMATECH and CNSE, to enable the development of advanced PV-related manufacturing processes throughout the United States

Other successful industry partnership models for consortia have been demonstrated in the medical fields of oncology [49] and dermatology [50] and were established in the interest of providing better healthcare capabilities through partnerships for technology development. Physicians have collaborative relationships with industry through consulting, sponsored research programs, joint development agreements, and technology development [49, 50]. Device companies, patients, academic researchers, and insurers gain benefits from the faster speed and lower costs of product development, and from the reduced risk and uncertainty of product development and implementation into the field [49], which often require collaborative standards development. These relationships also offer new opportunities and challenges for physicians seeking intellectual development. While physicians know medicine and science, other skills in technology development, testing, evaluation, or transfer may be needed, which is why these collaborations can be beneficial for all partners [50].

A recent, UV industry–driven partnership was formed between the IUVA and the European Photonics Industry Consortium (EPIC).^5^5 EPIC is an industry association that promotes the sustainable development of organizations working in the field of photonics in Europe. The association is composed of members from across the entire value chain from light-emitting diode (LED) lighting to photovoltaic solar energy, photonic integrated circuits, optical components, lasers, sensors, imaging, displays, projectors, optic fiber, and other photonic-related technologies. See https://www.epic-assoc.com/about-epic/ for more information. In 2020, both parties signed a collaboration agreement to strengthen the photonics industry on an international level, bringing together members and knowledge to better serve the industry for greater opportunities for all.^6^6 See https://www.novuslight.com/international-ultraviolet-association-and-epic-sign-mou_N10620.html for more information. This collaboration is targeting the development of cooperative activities. These include collaborative events, information exchange and promotion, and advisory developments to support best practices, with an emphasis on communication and interoperability between different parts of the UV technologies industry such as end users, service providers, equipment vendors and manufacturers, and technology developers. These efforts will also involve industry engagement for standards development, which will interface with metrology solutions, for more efficient and sustainable industry capabilities.

#### 3.3.2 NIST Examples

A recent example of a consortium focused on developing new metrology where NIST was involved is the NIST Rapid Microbial Testing Methods Consortium (RMTMC; Table 6) [51]. This consortium was launched in September 2020 to address the need for measurements and standards, including reference materials, to increase confidence in the use of rapid testing for microbial contaminants in regenerative medicine and advanced therapy products, but it is also applicable to UV device manufacturers wanting to enter the healthcare market. Any advances in microbial testing methods to ensure fit-for-purpose safety assessments for product development could be extremely useful to the UV device industry seeking fit-for-purpose materials or best practices for confidence building and assurance evaluation in efficacy testing protocols. The benefits to participants are clear: There are options for stakeholders to provide input into the design of microbial reference materials, as well as to actively design and participate in interlaboratory studies. Interlaboratory studies are essential for developing best practices and standard test methods. By participating in the RMTMC, participants will have access to improved testing capabilities.

**Table 6:** NIST Rapid Microbial Testing Methods Consortium (RMTMC) [51].

MODEL
• Convenes industry, academia, and government to identify and address measurement and standards needs across the rapid microbial testing methods field
• Enables members to work with NIST to develop measurement solutions and standards
• Leverages NIST expertise in measurement science, standards development, reference materials, technology development, and basic research
• Collaborates with related programs at other organizations
WHY NIST?
• Cross-disciplinary expertise in engineering and the physical, information, chemical, and biological sciences recognized internationally
• As a nonregulatory agency of the U.S. Department of Commerce, NIST does not impose standards; standards are accepted by consensus

The importance of NIST engagement in collaborative models supporting new metrology needs was recently highlighted by the Undersecretary for Standards and Technology and NIST Director [52] and the NIST Associate Director for Laboratory Programs [53] at the October 2020 Visiting Committee on Advanced Technology (VCAT)^7^7 The VCAT reviews and makes recommendations regarding general policy for NIST, its organization, its budget, and its programs, within the framework of applicable national policies as set forth by the president and the congress and submits an annual report to the Secretary of Commerce for submission to the U.S. Congress. public meeting [54]. Progress towards achieving the vision for the Return on Investment (ROI) Initiative [55] was highlighted [52].

As part of the ROI Initiative, private-sector engagement is important for NIST (Table 7). NIST deploys many different mechanisms to dynamically help industry with solutions to metrology needs (Table 8). Other examples of NIST-led partnership models solving metrology needs are provided in Sec. 4.1.3 and demonstrate the importance of shared resources and facilities.

**Table 7:** NIST and private-sector engagement through the Return-on-Investment (ROI) Initiative strategy^a^ to increase engagement with private-sector development experts and investors [52, 55].

Target	Examples of Progress
• Improved clarity and use of best practices by federal laboratories via streamlined partnership agreements	• Proposed legislation and regulation updates for “speed-of-business–based” best practices and tools for technology transfer that deliver government-wide modern, streamlined, and responsive customer experiences
• Increased private-sector collaborations for translational research and development via expanded partnership agreements	• Proposed legislation to support uniform, government-wide translational research and collaboration by simplifying, accelerating, tailoring, and executing partnership agreements
• Accelerated federal laboratory technology maturation via private-sector investment through nonprofit foundations (noting existing legislation does not provide all federal agencies the ability to establish these)	• Encouraged the development of uniform capabilities for federal agencies to establish nonprofit foundations to advance the accomplishment of agency missions by attracting private-sector investment

^a^ This is one of five strategies in the ROI Initiative to improve the transfer of technology from federally funded research and development to the private sector to promote U.S. economic growth and national security [55].

In addition to the mechanisms listed in Table 8, NIST also formally collaborates with industry, academia, and other government agencies to perform research that furthers the NIST mission through cooperative research and development agreements, the NIST Guest Researcher Program, and research grants through federal funding opportunities. NIST researchers frequently collaborate informally with researchers at other organizations. These collaborations often result in joint peer-reviewed papers, short-term visits, and sharing of research methods.

Cooperative research and development programs are generally with nonfederal entities and mainly U.S. industry partners. Cooperative research programs are formalized under a Cooperative Research and Development Agreement (CRADA) subject to being acceptable to NIST approval authorities. Research work under a CRADA may be performed at NIST, at the laboratory of the nonfederal collaborator(s), or at both institutions. Work is usually supported by contributions from all partners in the CRADA. NIST contributions to the CRADA can take the form of personnel, facilities, equipment, and other resources, but NIST cannot provide funds to the other collaborator(s). Collaborator(s) contributions can take the form of funds, personnel, facilities, equipment, and other resources.

The NIST Guest Researcher Program provides access for technically qualified scientists to NIST facilities and equipment while working with NIST staff on projects of mutual interest while being supported by their home institutions. Research results are available to the public. If confidentiality of cooperative research results is desired, a CRADA may be more appropriate. The NIST Guest Research Program supports domestic and foreign scientists.

NIST also offers research grants through federal funding opportunities that vary with each fiscal year. NIST federal funding opportunities support various disciplines of measurements, science, and engineering research programs.^8^8 See www.grants.gov for a current listing of federal funding opportunities. A grant or cooperative agreement is not the correct funding vehicle if the principal purpose is to provide products or services for the direct benefit or use of the federal government. Rather, a contract opportunity is better suited for such products or services. Contract opportunities are procurement notices that offer options of doing business with the government. Opportunities include presolicitation notices, solicitation notices, award notices, and sole source notices.^9^9 See www.sam.gov for a current listing of federal contracting opportunities.

**Table 8:** Mechanisms NIST uses for engaging with stakeholders [53].

Engagement	Benefits and Challenges	Selected Examples
Frameworks: Voluntary frameworks developed with extensive stakeholder engagement provide standards, guidelines, and practices	Benefits: • Target community is motivated to participate, has resources, and is organized • NIST can help drive communities forward faster Challenges: • NIST must have technical depth and respect in the community • May detract efforts from research to convening roles • Increasingly becoming a role that key stakeholders expect	Cybersecurity Framework: Helping organizations to better understand and improve management of cybersecurity risk. Community Resilience: Helping stakeholders prepare for anticipated hazards, adapt to changing conditions, and withstand and recover rapidly from natural, technological, and human-caused hazard events. Big Data Interoperability: Public working group building a consensus-based framework for a vendor-neutral, technology- and infrastructure-independent ecosystem to process and analyze ever-increasingly large data sets in any computing environment.
Consortia: Public-private partnerships nurture a community and identify shared challenges	Benefits: • NIST aligns research programs and measurement services with community needs • Access to small and large companies Challenges: • Stakeholder expect that NIST will address key needs • Continued management of agreements and funding structures is required to maintain some consortia	nSoft Consortium: Public-private consortium that enables U.S.-based manufacturers to learn about and access neutron tools to solve problems in manufacturing (see Sec. 4.1.3). Quantum Economic Development Consortium: Consortium of stakeholders that aims to enable and grow the U.S. quantum industry as part of the federal strategy for advancing quantum information science. Genome Editing Consortium: Public-private consortium working to address the measurements and standards needed to increase confidence and lower the risk of utilizing genome editing technologies in research and commercial products.
Prize Competitions: Prizes are awarded competitively to stimulate innovation and advance the NIST mission	Benefits: • Establishes ambitious goal without predicting who is most likely to succeed • Reaches more R&D community members • Inspires risk-taking by offering a level playing field • Challenges: • Requires prize competition expertise and infrastructure • Sustaining the community and follow-on investments after competition ends	NIST has offered over 21 challenges with over $4,000,000 awarded in prizes, many in the Public Safety Communications Research Division in 2020, for example, the CHARIoT Challenge to build augmented reality interfaces or internet of things data emulators with award prizes and technical services valued up to $1,100,000 [56]. Other NIST challenges include Head Health Challenge III offered by NIST, the National Football League, Under Armour, and General Electric to support the discovery, design, and development of advanced materials that better absorb or dissipate impact through a national competition offering up to $2 million in prizes [57].

## 4 General Considerations for Building Collaborative Models in the UV Industry

**Fig. 5:**
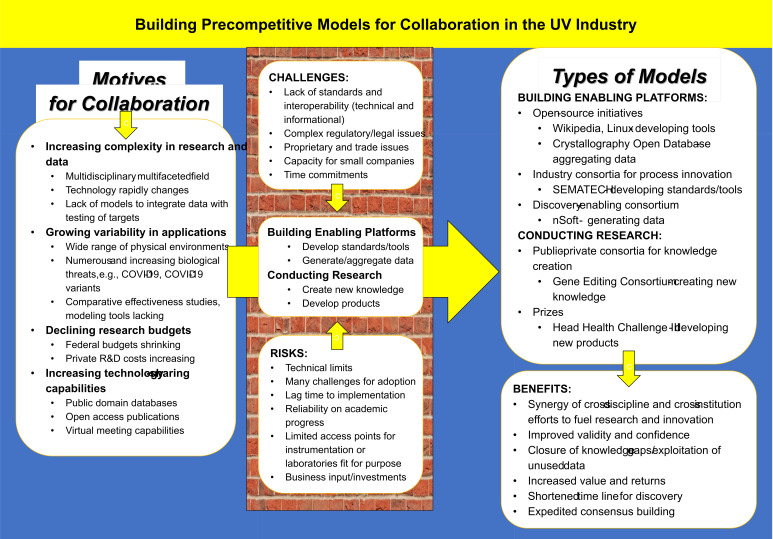
Possible models for precompetitive collaboration as applicable to the UV industry with examples showing risks, challenges, and benefits, adapted from the Institute of Medicine Extending the Spectrum of Precompetitive Collaboration in Oncology Research: Workshop Summary [49].

The National Cancer Policy Forum of the Institute of Medicine (IOM) closely examined why it is necessary, and what is needed, to build and sustain precompetitive collaborations in oncology research for the benefit of all participants [49]. The concept of precompetitive collaboration is neither new nor limited to biomedical applications. For example, the software industry is also known for its precompetitive collaborations, which has been defined as “competitors sharing early stages of research that benefit all” [49, 58]. However, challenges arise due to the complexity of such organizations. These include defining the research agenda and choosing projects; transferring research results to participants; transferring technology; and adapting to change in economic and technological environments [47]. Therefore, it is essential to consider the different complex motives for building collaborative models (Fig. 5). These are described below in the context of the UV industry in Sec. 4.1 and Sec. 4.2; Sec. 4.1 includes a discussion of motives specific to the UV industry to create collaborative models and a review of selected successful NIST collaborative models that could be applicable to the UV industry using shared resource facilities. Section 4.2 provides a short overview of precompetitive collaborations that would be applicable to the UV industry and the motives described in Sec. 4.1.

### 4.1 Motives for Precompetitive Collaborations

**Fig. 6:**
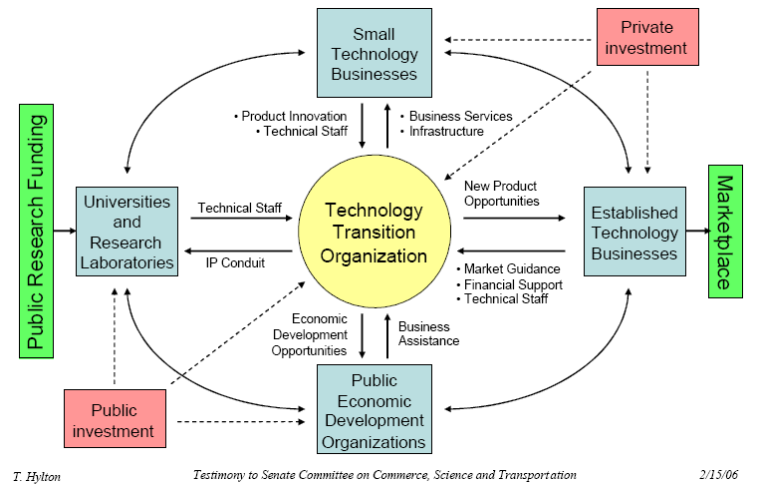
The “Hylton” model of facilitated technology transfer [59]. IP is “intellectual property”.

It may be expedient to create an industry-specific consortium to engage with the government in a public-private partnership (PPP). The consortium could serve as a technology transfer organization to facilitate the transfer of enabling technology from government laboratories to industry, manage market strategy coordination and roadmapping, as well as referee the transfer of innovation technologies from small startup companies to well-established companies to accelerate the market introduction of emerging technologies [59] as shown in Fig. 6.

#### 4.1.1 Increasing Complexity in UV Disinfection Research and Data

There is an ever-growing complexity of basic and applied research in UV technologies for infection prevention and control. The problems and solutions are multidimensional. The UV radiation source matters, *e.g.*, gas discharge (low-pressure or medium-pressure mercury lamps, xenon), solid state (UV LEDs), others (lasers, pulsed sources). Also, the wavelength matters (single or multiple wavelengths, broad spectrum), and there are various regions to consider, such as UV-C (200 nm to 280 nm); far-UV (207 nm to 220 nm); and near-UV (≈ 407 nm); see Sec. 2 in Ref. [6] for a short background on UV radiation. There has been an increased interest in finding additional wavelengths of interest from the vacuum-UV region (100 nm to 200 nm) to violet light (>400 nm) that may produce antimicrobial benefits [6]. A consortium model would help to accelerate research and development to investigate such multidimensional problems, including exploring the use of the different wavelengths of energy for disinfection applications.

**Fig. 7:**
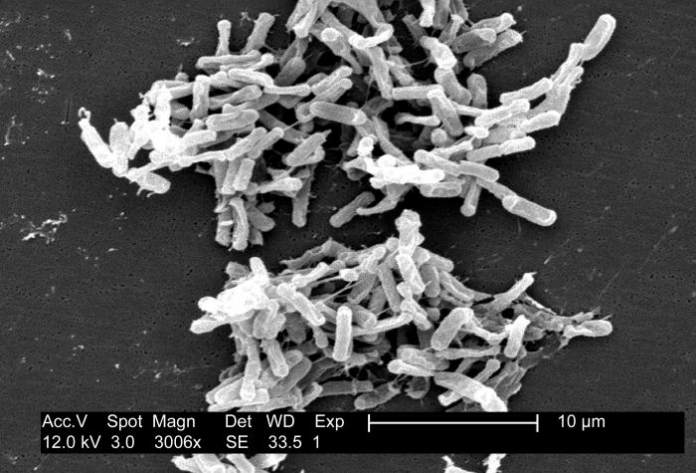
Scanning electron micrograph of *Clostridioides difficile* as described in the text (field of view ≈ 40.3 µm) [61].

Measurement of biological efficacy also is not UV source or target specific. There is large variation in UV inactivation values in the literature, *e.g.*, 67,567 μW⋅s/cm^2^ to 342,667 μW⋅s/cm^2^ for *Clostridium difficile* (now referred to as *Clostridioides difficile* or *C. difficile*; Fig. 7) at 3 log_10_ reduction^10^10 3 log_10_ units refers to a 99.9% reduction, calculated as log_10_(*N*_0_/*N*), where *N* is the initial value, and *N* is the final value. or 99.9%) [3, 60]. In Fig. 7, a scanning electron microscope image depicts a large grouping of rod-shaped, Gram-positive *C. difficile* bacteria [61]. The organisms in the micrograph were obtained because of an outbreak of gastrointestinal illness and cultured from a stool sample. Note the clustering (clumping) of these bacteria. Clustering can impede UV irradiation and contribute to the variability in required dose [62]. It has been shown to have an extrinsic photoprotective effect [63]. Recent mathematical models describe this phenomenon [63], further demonstrating the increasing complexity in research and data needs for UV disinfection and the indispensability of predictive and modeling capabilities to better enable applications and innovation (as was suggested at the NIST workshop [6]). In 2019, the U.S. Centers for Disease Control and Prevention (CDC) estimated there were 223,900 cases in hospitalized patients and 12,800 deaths in 2017 due to *C. difficile*, with estimated attributable healthcare costs of $1 billion [64]. While HAI *C. difficile* cases are decreasing, community-associated cases are not. Strategies to reduce *C. difficile* infections include improving antibiotic use, infection prevention, and healthcare facility cleaning and disinfection, all of which add to the complexity in UV disinfection research and data [65, 66].

Additional factors contributing to the complexity in research and data are the duration of exposure to energy and distance from the energy source. There is an increasing need to integrate the rapidly evolving types and amounts of biological materials and patients and test methods with the physics (radiation dynamics) and chemistry (bio-, physical-, and photochemistry) into new technology development. This requires big data approaches with data repositories and reliable and sophisticated data architecture to capture and analyze data with automated approaches [6]. The development of modeling or simulation tools, such as the clustering model mentioned previously [63], is needed to understand and predict pathogen biology, movement, and inactivation in combination, as was recommended at the NIST workshop [6]. Modeling and simulation tools are even more needed to help understand the application of UV for inactivation of microorganisms, including SARS-CoV-2 and its variants, in environments beyond healthcare facilities, such as for public buildings and in public transportation (air, rail, and motor vehicle).

#### 4.1.2 Growing Variability in UV Applications for Healthcare

The numbers and types of physical targets for disinfection by UV technologies are rapidly growing and changing. For example, in a hospital patient’s physical environment, there is a large variation in the number and type of physical items and surfaces that vary in scale. Classes of items, each with various numbers of items within, include furniture, equipment, technology, and architectural features in the layout (windowsills, windows, curtains, or handrails). Surface types include both soft, porous surfaces, such as curtains, and hard, nonporous surfaces, such as bed rails and medical equipment, which can be large or small in scale [67, 68]. Comparative effectiveness studies will be essential for understanding the most appropriate dose-response for the whole-room disinfection approach of a patient’s environment and to map out operational schema needed by hospital management. A wide range of radiation standards is available, but the needs are growing [69]. Air becomes a separate element in this endeavor, and it has a whole different set of variables and dimensions, ranging from the micrometer scale of aerosols to the macro-industrial meter scale of air-flow ducts in a building [70]. Current infrastructure needs to consider sophisticated modeling techniques with human-environment interactions factored in to approximate realistic indoor air-flow patterns and pathogen-movement patterns in hospitals [71]. Mousavi [71] is investigating architect perspectives on improving hospital design for the use of light to treat HAIs. This involves creating capabilities to predict contamination probability beyond single-room geometry. This speaks to the importance of cross-discipline engagement for any collaborative approach supporting UV technologies for healthcare.

As the number of physical targets grows, so too do the number of biological targets. In 2019, the U.S. CDC became concerned about rising infections resistant to antibiotics, which threaten the progress made to protect patients from HAIs in healthcare [64]. Currently, 18 antibiotic-resistant bacteria and fungi threats are considered either urgent, serious, or concerning to humans (Table 9). There is also a “watch list” with three targets. These have not spread resistance widely in the United States but could become common without a continued aggressive approach to study and understand their complexity and how they evolve. Given their complexity, cross-disciplinary collaborative approaches to manage and reduce these threats to a point where they are considered rare will be essential.

**Table 9:** Current bacteria and fungi posing antibiotic-resistance threats [64].

Urgent	Serious	Concerning & Watch List
• Carbapenem-resistant o *Acinetobacter* o *Candida auris* o *Clostridioides difficile* (see Fig. 7) • Carbapenem-resistant Enterobacteriaceae • Drug-resistant *Neisseria gonorrhoeae*	• Drug-resistant *Campylobacter* • Drug-resistant *Candida* • Extended-spectrum beta-lactamase (ESBL)-producing Enterobacteriaceae • Vancomycin-resistant *Enterococci* (VRE) • Multidrug-resistant *Pseudomonas aeruginosa* • Drug-resistant nontyphoidal *Salmonella* • Drug-resistant *Salmonella* serotype Typhi • Drug-resistant *Shigella* • Methicillin-resistant *Staphylococcus aureus* (MRSA) • Drug-resistant *Streptococcus pneumoniae* • Drug-resistant tuberculosis	Concerning Threats • Erythromycin-resistant Group A *Streptococcus* • Clindamycin-resistant Group B *Streptococcus* Watch List • Azole-resistant *Aspergillus fumigatus* • Drug-resistant *Mycoplasma genitalium* • Drug-resistant *Bordetella pertussis*

#### 4.1.3 Declining Research Budgets: Shared Resources and Facilities Offer Solutions

In the United States, the federal government is the largest funder of academic research and development. In 2018, six agencies provided more than 90% of support for academic research and development, with the Department of Health and Human Services, largely through the National Institutes of Health (NIH), providing more than half of that. However, after a long period of increase, federal support for basic research at academic institutions has declined over the last 10 years [72]. For fiscal year (FY) 2021, the NIH budget request proposed a research program level of $39.1 billion, a 6.1× decrease from the FY 2020 program level (regular appropriations). In contrast, private-sector investment in research and development has had the opposite trend over the past decade, while maintaining a level that is more than double the federal amount. Declining research budgets are a motivating factor to create models for shared resources and facilities [34, 59].

**Fig. 8:**
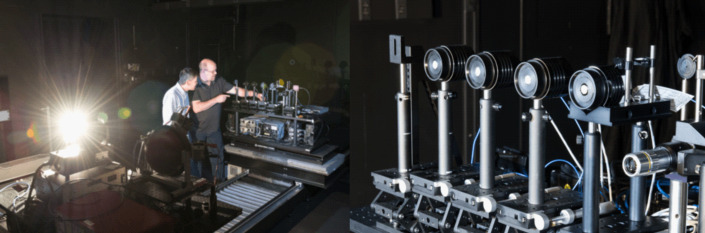
Left: NIST’s Yuqin Zong and Cameron Miller in the new, fully automated NIST photometry laboratory. Right: A row of identical photometers (light detectors) sitting on one of the new automated equipment tables in the NIST laboratory. Multiple identical detectors are used for each measurement so that researchers can ensure the readings are accurate [73]. Credit: Jennifer Lauren Lee/NIST.

Private-sector research and development tend to cost more than federal research and development, with the work focusing less on research and more on development. Development requires major infrastructure, facilities, and equipment that increase investments. Typically, the federal government invests in specialized facilities that focus on fundamental research. Private-sector investments can be leveraged by federal investments in specialized facilities through consortia approaches such as those described in Sec. 3. These facilities are typically not affordable by industry alone and can be financially straining to maintain and staff. For example, NIST’s photometry laboratory (Fig. 8) provides specialized radiation source calibration facilities [73] that would not be possible for one entity to support, maintain, and staff by itself. As one example, the laboratory has been meeting the industry’s needs by offering an LED brightness and photometer calibration service.

A recent update to the NIST photometry laboratory makes the NIST LED brightness and photometer calibration service “one of the best—if not the best—in the world” [73] with measurement uncertainties of 0.2% or less. Plans are under way for adding a goniophotometer to offer a new type of service: measuring the LEDs’ UV output [73]. This is important because recent research suggests UV LEDs with peak emission at approximately 286 nm could serve as an effective tool in the fight against human coronaviruses [74], and new modeling tools are helping to predict UV-C LED irradiance distributions on surfaces [75]. With a potential large increase in industry needs for UV-C LED output measurements, the possibilities for delivering both technology and expertise to industry through a consortium are considerable.

**Fig. 9:**
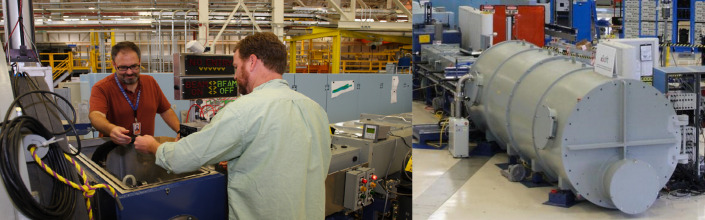
Left: Researchers work collaboratively at the NIST Center for Neutron Research as part of the nSoft Consortium. Right: The 10 m small-angle neutron scattering instrument accessible to members of the consortium. The technique enables structural observations to be connected to processes used in manufacturing, including shear, high pressure, high temperature, and environmental exposure. It is foundational for the development of structure-property-performance relationships in solid materials, liquids, and mixtures [76, 79]. Credit: NIST.

The NIST nSoft Consortium listed in Table 8 is an example of a model that was built around transferring capabilities and expertise, which is also applicable to the UV industry. Members gain access to specialized neutron facilities and expertise for neutron-based measurement science, such as the 10 m small-angle neutron scattering instrument to measure correlations in spatial structure within solid and liquid materials (Fig. 9). Member companies participate in publicly accessible research led by NIST staff at the NIST Center for Neutron Research and the NIST laboratories while leveraging NIST’s unique expertise to develop new capabilities and create data that would otherwise not be available to them in isolation [76]. Examples of progress for nSoft members are new methods for measuring the penetration of boron-containing materials into synthetic skin [77] and a novel noninvasive method to directly quantify surface heterogeneity of porous materials [78]. The impact of the consortium comes from close and extended collaboration, where member companies often have staff on the NIST campus for extended periods of time. Members, having participated on-site at the NIST Center for Neutron Research, gain expertise in the use of equipment and software and network with other participants through training classes, research seminars, and annual meetings, where new technical developments and existing capabilities can be shared. This is an example of the dynamic process stream among government, academia, and industry outlined by Young [29] and shown in Fig. 3.

#### 4.1.4 Increasing Technology-Sharing Capabilities

In today’s electronic age, greater access to public domain databases and open-access publications from virtually anywhere has increased the ability to form collaborative models. Not only has the quantity of information grown in the technical world, but the quality of data has improved, with more open-source tools for data analysis and use, such as those being developed through the Big Data Interoperability Framework mentioned in Table 7. The IOM workshop (see Sec. 4) for collaborative models in oncology noted: “What is available in the commons is starting to be almost as good as what companies can develop themselves internally” [49].

At the NIST workshop [6], a consistent message was that data availability in the public domain is an essential tool for the burgeoning UV research and development community supporting healthcare. As the various types of UV sources and number of pathogens continue to grow, and performance metrics of photometer radiometers become more advanced, common data platforms with shared data and tools for modeling and simulations are increasingly important with respect to open-source initiatives, as discussed in Sec. 4.2.

### 4.2 Types of Precompetitive Collaborations

The factors described in Sec. 3 are examples of motivation for forming collaborative partnerships to help specialized industry sectors achieve common goals. For example, in the field of oncology, collaborative partnerships are necessary for better oncological treatment to combat cancer and prevent deaths [49]; a similar end goal for the UV industry is discussed in Sec. 1. As noted above, precompetitive collaborations enable the development of common sets of data and tools that can lead to standards for the community and the needed scale for research. Enabling platforms include process innovation models (such as SEMATECH discussed in Sec. 1 and Sec. 3.2.1), discovery science platforms (such as the NIST nSoft Consortium discussed in Sec. 4.1.3), and open-source initiatives.

Open-source initiatives are those that are open to the public and continue to lead to new tools for public use. Familiar examples include Wikipedia or Linux open software. Databases can be open source as well, such as the NIST Genome Editing Consortium (Table 8; [80]). This is a compilation of open genome editing research and materials in one place for community access and efficiency. As a result of this open source of data, the development of standards and measurements for targeted genome editing is possible and is leading to better capabilities in healthcare. A second example of an open-source database is the NIST Ballistics Toolmark Research Database [81]. This database provides the most up-to-date bullet and cartridge case toolmark data, reflectance microscopy images, and three-dimensional surface topography data that are submitted by the community to support forensics science research. All these enabling platforms could be applicable to the UV industry.

Prize challenges could also be applicable to the UV industry (see Table 8; Fig. 5) and could encourage innovation and new products through shared competition options. Prize challenges are not research intense but rather end-goal driven to develop a product. Prize challenges in the UV industry could result from crowd-sourced funding through project calls to develop novel devices, large-scale equipment, or information technology platforms applicable to infection reduction.

While there are many models for collaboration that are applicable to the UV industry, the SEMATECH model seems most applicable, given the comparable global scope and goals to enable greater benefits for all participants. In the final section of the paper (Sec. 5), we propose a conceptual organizational model using the SEMATECH principles, as well as those from the Semiconductor Research Corporation (SRC) [82, 83] as applied to the UV industry. This structural model for collaboration has been shown to be an effective organizational structure supporting the principle of cooperative government-industry research activity that has been adopted and accelerated—often with success—in other semiconductor-producing countries and regions [10, 45–48]. The model has also been discussed as a fundamental example for precompetitive collaboration in other industries, such as oncology [49].

## 5 Conceptual Organizational Model for a Collaborative Partnership in UV Technologies for Healthcare: The BlueVioletTech Consortium

A UV-C stakeholder consortium could identify multi-industry needs, assess existing research and identify gaps, communicate these gaps to funding agencies, and establish collaborations with universities to work on solving the identified problems. Other federal laboratories, member companies, and existing consortia or partnerships, such as the IUVA-EPIC partnership (see Sec. 3.3.1), could also help through direct funding, technology transfer, and potentially in-kind efforts. While the roles and responsibilities of members and collaborative partners would need to be defined, all entities would work toward the same high-level goal: to enable better global health through ultraviolet radiation with innovation, education, and collaboration. It is envisioned that consortium members would have access to research results and knowledge of projects, but other consortium members, such as liaisons, could have access to more summarized, high-level project reviews, progress reports, or selected data modules. This model is very similar to the SRC model, which connects its members with academia (2400 university researchers), government partners, and 4700 industry liaisons [82]. In SRC, liaisons are member-company employees who join one or more SRC research efforts. By doing so, they aid and reap benefits for their companies in terms of early transfer of useful findings and technology [83]. This is an example of applying the SRC approach to better connect members and offer greater benefits to members in a UV industry consortium.

Conceptually, such an example precompetitive consortium organization (*e.g.*, a hypothetical “BlueVioletTech Consortium”) could draw from stakeholders identified in Table 1 to tie together the healthcare entities associated with the government, academic, and industry infrastructure development organizations (Fig. 3). A first step would be to agree on an agenda (see Table 10). Ideas for an initial agenda could be dedicated to addressing and facilitating common issues such as safety, testing, standardization, effective commercialization strategies, and education on disinfection utilizing UV radiation with direct application to HAIs and their eventual elimination. The roadmap in Table 3 could be a start for the effort. Expansion into other areas where UV disinfection is utilized would be anticipated to build a sustainable platform. Some examples of roles an industry organization could undertake in a UV consortium are:

(1) engaging membership-designated leads to utilize the capabilities and facilities of industry organization’s members through the consortium for cooperation, coordination, information sharing, and research and development projects;
(2) engaging industry and other stakeholders (Table 1) in projects undertaken by the consortium; and/or
(3) developing and supporting a platform for the consortium to manage the consortium and the daily needs for its operation.

See Table 10 for more information about the types of activities that are necessary for developing and managing a consortium.

BlueVioletTech could, as an example, be led by an elected governing council, with technical direction provided by a technical program committee (TPC) and a research advisory group. Benefits to members could be access to integrated assessments of research needs; periodic and annual progress reports; technical assessments of relevant research, databases, and modeling capabilities; and pooled resources for funding opportunities for collectively identified projects (see Sec. 5.2 for a description of possible funding structures, which could include members paying a fee to support resources available for projects). The consortium could also provide shared access to other existing or even new collaborations in and outside of the consortium as they become available, offering companies an opportunity to be relieved of their individual burden and to be connected or to network with other relevant entities. The consortium could work with researchers to grow technology and capabilities and improve access to existing capabilities that will benefit the membership and participating parties on all levels as defined by the organization. Based on the examples provided in Sec. 3 and Sec. 4, no one is left out in these types of scenarios; demonstrated successes offer substantial evidence for this: Participants on all levels gain some benefit and contribute to the greater goals of the organization.

At the operational level, BlueVioletTech could, as an example, initially focus on UV sensors, performance standards, metrology, materials, chemistry, and measurements needed to assure bioremediation aspects of the technology through its project calls. The fundamental concepts are to utilize the government, academic, and industry-wide knowledge bases and resources; identify and address industry-wide obstacles and capacity-building needs, as well as conduct cost and supply chain optimization for the consortium; and rapidly transfer technology from research universities and government laboratories to the product development stage [84]. Input from federal and academic technology transfer programs would be essential to the application of this concept.

The technology developed in the consortium could support the entire UV industry because many of the problems researched are pervasive. For example, the consortium could support the growing segment of a total global UV market for HAI-related UV disinfection equipment. The UV disinfection equipment market size is projected to reach $9.2 billion by 2026 from an estimated $4.8 billion in 2021 [11]. Growth factors include the use of UV disinfection equipment in healthcare facilities for disinfecting the hospital environment and increasing demand to combat SARS-CoV-2 and variants, as well as the growing LED market [11]. In addition, the reliability of UV disinfection equipment with UV lamps is expected to influence the market further. While water, wastewater, and process water applications are currently the top three applications for the UV equipment market, the importance of air and surface applications is rising due to the COVID-19 pandemic; all applications will benefit.

It is important to note that consortia such as the one proposed here are not set up to address company-specific applications of the developed technology; hence, the consortium body is typically not involved in regulatory issues (which tend to be application specific). However, the consortium can offer ways to provide information-sharing opportunities, such as webinars or the creation of task forces, for discussions about important regulatory needs and challenges and potential ways to overcome those challenges through a consensus-building approach, with the major input coming from the consortium members. The consortium would then be responsible for disseminating that information to its members and affiliates through newsletters, email communications, membership meetings, or other approaches.

In addition, consortia do not provide operational validation services to its members, but rather nonmember participants, who join in the discussions, may become third-party providers for those types of capabilities, and they will then incorporate the testing methods developed through consensus. Operational validation of any commonly developed technology concept would need to be completed with noncommercial prototypes and demonstration projects. This is not uncommon in consortium models such as the ones described in this paper. Also see Sec. 2.2 for more information on the role of accreditation bodies, who could be eligible to be consortium members, but their role would not be to develop the accreditation processes. Rather, the consortium members would develop those through voluntary consensus-building approaches with standard development organizations and the UV community.

### 5.1 Fundamental Research

By drawing all the experts together into a consortium such as BlueVioletTech, transformational advances through research and development are possible. For example, fundamental research is needed in basic photochemistry, photophysics, and photobiology, and these topics need to be central to academic curricula in engineering, materials science, and biology, as was defined at the NIST workshop [6]. Optics and photonics technologies and their integration for a variety of commercial-scale disinfection applications are additional examples, along with UV-C data and modeling [6]. BlueVioletTech-led platforms that house projects in these areas could be successful at accelerating improvements in existing technology and development of new technology. Equipment and instrumentation developers would be able to take advantage of shared research and resources for achieving designs to generate, propagate, control, and detect light in healthcare settings. Biologists and medical experts can participate to achieve system-level designs and development goals for preventing and eliminating infectious agents in healthcare settings. All collaborating parties could participate in continuous roadmapping exercises to meet technical challenges and grow opportunities. Immediate challenges could also be overcome through cooperative engagement of parties to solve the most pressing problems.

All collaborating parties would need to agree on what would be sharable, such as common data elements, information technology infrastructure (cloud-based data banks or modeling tools), clinical trials with common member participation, or standard language for contracts or agreements used to build collaborations. For example, the partnership intermediary agreements that were recently developed between the Maryland Technology Development Corporation and the U.S. Naval Surface Warfare Center became an award-winning standard for all future U.S. Navy agreements with a template that allowed the agreements to meet the needs of the individual laboratories [85].

### 5.2 Funding

An initial funding model may be limited to a partnership-based model based on dues, but additional funds could likely emerge once the organization is well defined, as demonstrated in the example cases previously discussed. Unlike the initiation of SEMATECH, continued federal fiscal concerns are forcing difficult decisions about the extent and types of research the government should support for achieving pathogen-abatement strategies. That could change. Whereas the current death rate due to HAIs is approximately 72,000 deaths per year in the United States [16], the number of deaths due to COVID-19 was many times greater in the United States in 2020 [86]. The potential application of UV to decrease or prevent transmission of COVID-19 through UV surface- and air-disinfection strategies is a compelling justification for entities to engage in a consortium.

For BlueVioletTech, one model is that dues-paying members would be able to vote in elections and participate in collaborations. Nonvoting members would include non-dues-paying universities, governmental organizations, research institutions, nongovernmental nonprofit organizations, and trade associations. A host for BlueVioletTech could be an existing nonprofit organization that provides infrastructure and support. Members could be offered opportunities that are tiered; for example, members in one tier could provide input to develop projects that are specific to the needs of the companies in that tier. This offers the advantage of providing customized approaches for specialized or niche companies who generally cannot raise capital for such projects but could benefit from the collective approach offered by the consortium and possibly solve a significant problem for the entire industry. For example, in SEMATECH, specialized cooperative research efforts evaluated the feasibility of forming ultrashallow junctions by three competing methods: (1) conventional ion implantation, (2) plasma source ion implantation, and (3) projection gas immersion laser doping. These led to results that assisted the consortium in making its 0.18 μm doping technology decision as required by the roadmap [87], among other successes (see Table 5).

Metrics for progress could be the number of voluntary standards and test methods developed for UV healthcare equipment. Database platforms and publications are also metrics for progress. Metrics for success could include the numbers of instrument improvements, infections prevented, and lives saved, and medical costs averted through HAI prevention and treatment.

### 5.3 Communication and Perception Strategies

It is essential for BlueVioletTech to include the development and use of communication and perception strategies to raise awareness about the consortium’s programs and events and, equally important, UV technology. A communications strategy should include a program manager and team for media such as websites, e-mail marketing, social media, and other channels for disseminating technical and general information. Communications will amplify messaging about the benefits and use of UV and help prevent disinformation campaigns that negatively portray UV risk or hazards. The strategy should consider including elements for educational professionals too, with a comprehensive approach that is responsive to the needs spanning younger (preschool) to older generations with information ranging from that for the public to lesson plans and degree curricula. For example, literature and media materials developed should consider topics such as:

• why UV technology is unique and special;
• basic information about UV sources and their use; and
• an introduction of UV technology for children.

In addition, a communications strategy should include a program manager and team to convene events for conceptualizing, planning, executing, and evaluating innovative and economical activities that advance the organization’s mission. These events should include: Research project team meetings, conferences, webinars, workshops, symposia, expositions at trade or technical events, stewardship lectures with donors (if applicable), and special events such as member-driven outreach events at schools, fairs, libraries, and museums in rural and urban locations to communicate the value of UV technology. Events should engage local or regional industries with interest or use in UV and community leadership. The latter is important for garnering local or regional economic development leadership support and recruiting new members (see Table 10).

### 5.4 Establishing the Consortium

Common stages for establishing a consortium are listed in Table 10. Information is included on what to do at each stage and approximately how long each stage will take.

**Table 10:** Stages for developing and establishing a consortium.^a^

Stage	What to Do	Suggested Approaches	Time (Stages May Overlap)
Pre-startup scoping and planning by the community	• Scope the conditions and timing for establishment; gain consensus about the need for a consortium • Develop a list of founding organizations • Develop roadmaps of challenges and research and development needs • Develop a first draft vision and mission statement and define core values • Develop a plan for establishment and adoption of the organization by members and affiliates • Develop a plan for funding mechanisms	Hold workshops, panels, or special sessions at industry association meetings and virtual events (multiple types and numbers of events may be required) led by community organizations	3 months
Formation of working group	• Establish a group of individuals representing a broad range of organizations, each with delegated authority from their own organization to make decisions and drive the consortium development forward to: o Establish the founding principles of the consortium o Select an operational model and the composition of the consortium o Seek support of key regional or local economic leadership for the organizations participating o Develop core documents on goals and plans for the new organization and recruitment, communication, and collaboration strategies o Develop core documents for executive oversight	Hold a kickoff meeting followed by regularly scheduled meetings to do the work; may require a second workshop for business planning	6 months
Development of funding	• Establish a subgroup on funding and finance to: o Develop clear documentation for the amount of funding that is required o Identify potential funding sources	Hold regularly scheduled meetings to do the work	6 months and on
Stakeholder engagement	• Establish a subgroup on stakeholder engagement and recruitment to: o Develop a stakeholder map o Develop prequalification criteria for consortium members and affiliates o Develop roles and responsibilities of members and affiliates • Hold events to recruit and inform stakeholders • Keep key regional or local economic leadership engaged and recruit economic development organizations • Create formal processes for members to engage with laboratories and test facilities^b^	Hold regularly scheduled meetings to do the work plus special events	6 months and on
Set up central management hub with executive leadership and oversight	• Establish a central management hub, which could be through a trade organization, to run the consortium business, scope for projects, and grow the network of members and affiliates. • Continue support for the finance working group • Establish an executive advisory board to advise the central management hub	Establish a central management hub organization and recruit leadership and staff or outsource to members	9 months
Develop and open projects calls	• Develop project call topics to address immediate needs^c^ • Develop and adopt a bidding strategy for projects • Develop and adopt a strategy for managing internal competitions • Create a sub–working group to plan for internal disputes or arbitration • Create a sub–working group for long-term needs planning	Conduct work through hub team working with executive advisory board	1 year and on
Service delivery	• Provide project management oversight • Support members and project work • Support data management • Support networking and communications	Conduct work through hub team working with executive advisory board	1 year and on
Review, revise, refresh activities	• Plan events to evaluate progress and reaffirm organizational structure and policies • Evaluate roles and responsibilities of organization participants • Conduct roadmapping events • Continue recruitment • Maintain operations to achieve sustainability	Hold annual meetings and retreats for leadership and members	1.5 years and on

^a^ Adapted from Ref. [88].

^b^ For example, cooperative agreements with government facilities such as the NIST Photometry Laboratory (see Sec. 4.1.3 and Fig. 8).

^c^ Immediate needs are often assessed using information from scoping, planning, and stakeholder engagement events and other events convened by community stakeholders. An example of a community stakeholder workshop is the recent 2022 U.S. Department of Energy Solid-State Lighting Public Workshop [89]. A panel on germicidal UV highlighted current research and development needs for the germicidal UV industry, which included the development of standardized activation curves; development of standardized quality-control and quality-management programs for tracking variables in UV disinfection of air and surfaces; and developing capabilities for UV disinfection at new wavelengths of energy for disinfections, such as in the violet (>400 nm) region, where applications currently require very high doses not practical in an occupied environment [89, 90]. These are examples of needs that might not be identified at the onset of the consortium development but are later evident based on input from the community at events other than those sponsored by the consortium. See Sec. 4.1.1 and Sec. 4.1.2 for additional information about current research and development needs for the germicidal UV industry.

## 6 Summary

Expertise in the critical and acute research areas supporting pathogen abatement is found in many public- and private-sector entities. By bringing entities together to participate in a collaborative consortium, metrology can be advanced to support the measurements, standards, technology, and data needs of the global UV industry. A government, academia, and industry cooperation model, such as the hypothetical BlueVioletTech (BVTech) described in this paper, would support basic research on which the industry depends and enable a dynamic innovation process to uniformly meet challenges and grow opportunities for better public health. It could provide a platform for basic science, offer shared leadership, enable high-risk research not possible by one entity, and close standards gaps. Many types of consortia have been established at NIST and elsewhere in the public and private sectors. It is recommended these be examined to help with decision making in this area, as was discussed at the NIST workshop [6].

The major stakeholders are UV-C manufacturers and the healthcare community supporting patients. Fundamental technological progress towards infection control and prevention is possible through a consortium approach. Research solutions, technology transfer, and implementation through shared projects and resources are more easily obtainable by industry consortia, as no one company can offer solutions to all the challenges an industry collectively faces. We have reviewed how collaboration and partnering are powerful tools and described how these tools have been shown to be successful for many industries. A UV industry consortium will offer collective periodic roadmapping, precompetitive research, and technology solutions to meet current and future challenges in public health.
